# Impact of freezing temperature (T_fr_) of Al_2_O_3_ and molecular diameter (H_2_O)_d_ on thermal enhancement in magnetized and radiative nanofluid with mixed convection

**DOI:** 10.1038/s41598-021-04587-9

**Published:** 2022-01-13

**Authors:** Adnan, Waqas Ashraf, Umar Khan, Amnah S. Al-Johani, Naveed Ahmed, Syed Tauseef Mohyud-Din, Ilyas Khan, Mulugeta Andualem

**Affiliations:** 1 Mohi-ud-Din Islamic University, Nerian Sharif, Azad Jammu and Kashmir 12080 Pakistan; 2 Department of Applied Mathematics and Statistics (AM&S), Institute of Space Technology (IST), Islamabad, 44000 Pakistan; 3 Department of Mathematics and Statistics, Hazara University, Mansehra, 21120 Pakistan; 4 Mathematics Department, Faculty of Science, University of Tabuk, Tabuk, Saudi Arabia; 5 Department of Mathematics, Faculty of Sciences, HITEC University, Taxila Cantt, 47070 Pakistan; 6 University of Multan, Multan, 66000 Pakistan; 7 Department of Mathematics, College of Science Al-Zulfi, Majmaah University, Al-Majmaah, 11952 Saudi Arabia; 8 Department of Mathematics, Bonga University, Bonga, Ethiopia

**Keywords:** Engineering, Mathematics and computing, Nanoscience and technology

## Abstract

The dynamics of nanofluid by considering the role of imposed Lorentz forces, thermal radiations and velocity slip effects over a vertically convectively heated surface is a topic of huge interest. Therefore, the said study is conducted for Al_2_O_3_-H_2_O nanofluid. Mathematical modelling of the problem is done via nanofluid effective correlations comprising the influences of freezing temperature, molecular diameter and similarity transformations. The results for multiple parameters are plotted and provide comprehensive discussion. From the analysis, it is examined that Al_2_O_3_-H_2_O nanofluid motion drops by strengthening Lorentz forces. The temperature in the nanofluid (Al_2_O_3_-H_2_O) is improved by inducing viscous dissipation effects (Ec number), surface convection (Biot number) and thermal radiations (Rd). Moreover, the shear stresses at the surface decreased due to higher magnetic field effects and rises due to velocity slip. A significant rise in Local Nusselt number is observed due to thermal radiations and Biot effects. Finally, enhanced heat transport mechanism in Al_2_O_3_-H_2_O is examined than a conventional liquid. Therefore, nanofluids are better for industrial applications and the uses of conventional liquids are limited due to low thermal conductivity.

## Introduction

The investigation of thermal enhancement in the nanofluid (Al_2_O_3_-H_2_O) is a topic of interest in recent time and attained much attention of the researchers and engineers. These nanofluids have high thermal performance rate in which their thermophysical characteristics, molecular and nanoparticles diameter playing significant role. Analysis of the nanofluids saturated by Al_2_O_3_ nanoparticles became very popular due to their multiple applications in various industries. Therefore, Fluid dynamists focused their attention to examine the characteristics of various nanofluids under various flow scenario including finite or semi-infinite flow regions.

The dynamics of chemically reacting fluid by taking convective flow condition into account were determined by Chu et al.^1^. They obtained dimensionless version of the flow model via similarity transformations and a numerical technique (RK method coupled with shooting algorithm) is adopted for mathematical analysis. The decrement in the fluid motion due to imposed magnetic field effects and increment in the temperature against convective condition are determined in the analysis. Further, they reported that thermal radiations are useful for thermal enhancement in the particular model and mass transport rises due to stronger soret effects. The heat transfer enhancement in 3D flow driven by a stretchable sheet by considering varying thermal conditions is examined by Liu and Andersson^2^. They conducted numerical analysis for the fluid dynamics and provided the graphical results with comprehensive discussion against the various flow parameters. Moreover, they observed that boundary layer thickness drops against the reduction of the surface temperature or by increasing the local thermal performance rate in bilateral directions.

In 2016, Mahanthesh et al.^3^ presented numerical simulations for the nanofluids under the impacts of Lorentz forces by taking heat flux surface condition. The analysis was conducted over a bidirectional stretchable geometry located in cartesian frame. They investigated dynamics of the nanofluids composed by various metals and their additives. The salient features of the pertinent flow parameters on the fluid motion, temperature, walls shear stresses and local rate of heat transport are computed and provided a comprehensive discussion. From their study, it is evident that addition of the nano additives in the base liquids have high thermal transport rate and are useful for different industrial applications. The vital role of slippery surface on the flow of nanofluid over a stretchable sheet was determined by Haq et al.^4^. They reported combined effects of thermal and velocity slips along with the restriction of zero normal flux of tiny particles over the surface.

The autocatalytic chemical reaction significantly alters the dynamics of hybrid nanofluid. Therefore, keeping in view the significance of thermal transport in hybrid nanofluid, Gul et al.^5^ reported the study of colloidal suspensions comprising SWCNTs and MWCNTs. They used EG as a host liquid and taken the flow in permeable medium. They modified the energy relation by adding Fourier heat flux and used autocatalytic reaction in the concentration constitutive relation. From the keen observation, augmentations in the entropy generation are noted against the pertinent parameters. Recently, Ramzan et al.^6^ prolonged the analysis of nanofluid by taking troian and thompson flow conditions on the surface. The proposed model was handled numerically and displayed results against the parameters. Further, they validated the analysis by providing comparison with published work under certain restrictions on the colloidal model.

The investigation of entropy in the nanofluids is significant and gained much attention of the researchers and scientists in the recent time. Lately, Ishak et al.^7^ examined the entropy behavior in the nanofluid flowing over a cylinder. In order to enhance the fluid characteristics, they used Al_2_O_3_ nanomaterial in the host liquid. By using numerical technique, they plotted the significant results for entropy by varying the pertinent flow parameters over the desired domain. In 2020, Sajid et al.^8^ introduced the dynamics of a new type of fluid known as Reiner–Philippoff fluid. They modeled the problem by incorporating the influences of thermal radiations. They highlighted the effects of varying thermal conductivity and internal heat source in the constitutive model and used numerical technique to display the results. Further, to authenticate the study, they made a comparative analysis under various conditions on the flow model.

The study of williamson nanofluid by considering the influences of porosity parameter over a stretchable surface is conducted by Bouslimi et al.^9^. For novel analysis, they incorporated the phenomenon of resistive heating and chemical reaction in the constitutive model. From the study, they concluded that the skin friction and nusselt number rises by increasing the strength of imposed Lorentz forces. The inspection of boundary layer flow for Eyring-powell nanofluid over a stretchable surface was examined by Ibrahim and Gadisa^10^. The Galerkin technique is used to explore the fluid characteristics over the desired domain against the multiple parameters. Further, recent studies related to the dynamics of the nanofluids under certain scenario were described in^11–18^ and the studies described therein.

The heat transport in the second generation of the fluid (nanofluid) attained huge popularity of the researchers, scientists and engineers due to their ultra-high thermal performance. These fluids resolved the problems of the industrialists and engineers regarding to the heat transfer as much heat transfer is acquire to accomplish many industrialists and engineer’s problems. Therefore, a significant analysis in the context of heat transfer is added in the scientific literature through the conducted analysis. It is evident that different physical effects like thermal radiations, magnetic field, mixed convection and viscous dissipation significantly alter the fluid thermal behaviour.

From the cited literature, it is noticed no studies regarding the thermal performance in Al_2_O_3_-H_2_O under aforementioned effects are reported so far. Therefore, a research paradigm is organized over a vertical stretching surface fixed in Cartesin coordinates system. The flow configuration will be modeled in cartesian region via similarity transformations and a numerical technique along with shooting algorithm will be implemented. The results against the parameters are then plotted and provided a comprehensive discussion. Form the results, it is examined that Al_2_O_3_-H_2_O has ultra-high thermal performance rate under thermal radiations, mixed convection, viscous dissipation and magnetic field. These fluids would be beneficial for cooling systems and many other industrial applications. On the other hand, parallel results for the regular liquid are also plotted under the same physical quantities and observed that the heat transfer in the liquid is low due to which it has very limited industrial applications. Further, the study is validated via comparison with previously published work.

## Mathematical modelling

### Problem statement and geometry

The flow of water saturated by aluminum oxide nanomaterial is taken over a vertical stretchable surface. The magnetic field is imposed perpendicular along z-axis in the existence of thermal radiations. Further, ${\stackrel{\smile}{u}}_{w}=a{\left(x+y\right)}^{n}$ and ${\stackrel{\smile}{v}}_{w}=b{\left(x+y\right)}^{n}$ are the velocities and the strength of magnetic field in ${\varvec{B}}={B}_{0}{\left(x+y\right)}^{0.5(n-1)}$. Moreover, the flow is subject to the following assumptions:

- The flow is viscous and incompressible.
- The host liquid and the nanomaterial are thermally in equilibrium.
- No slip exists between them.
- The nanofluid flow is radiative.

**Figure 1:**
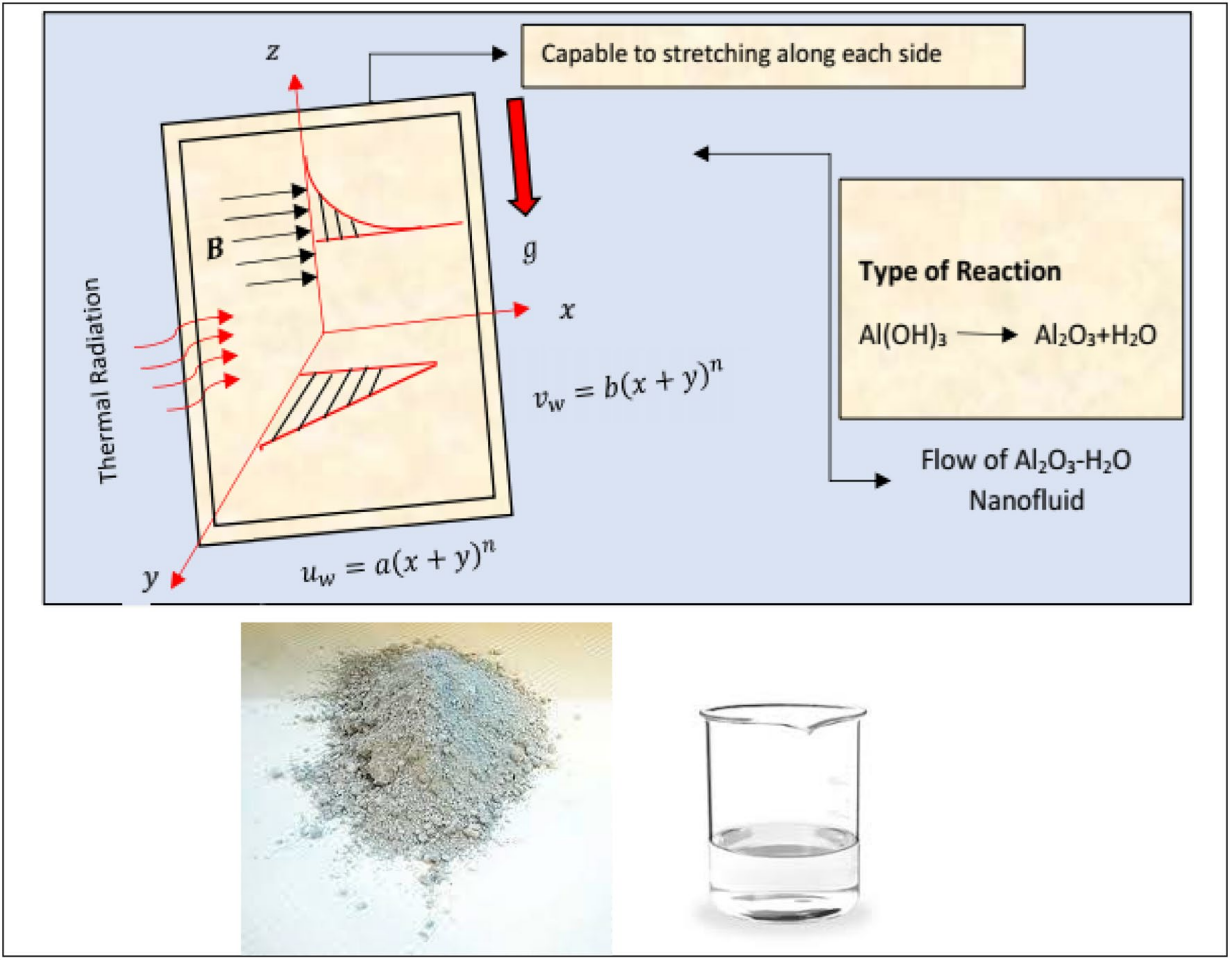
The flow scenerio of Al_2_O_3_-H_2_O.

Figure 1 depicted the flow configuration in the view of above assumptions.

### Nanofluid effective correlations

The effective nanofluid correlations significantly alters the nanofluid thermal characteristics. For under consideration nanofluid study, the following effective correlations comprising the influences of freezing temperature and molecular diameter are taken^19^:

1 $${\widehat{\rho }}_{nf}=\left[\left(1-\phi \right)+\frac{\phi {\widehat{\rho }}_{p}}{{\widehat{\rho }}_{f}}\right]{\widehat{\rho }}_{f},$$

2 $${\left(\widehat{\rho {C}_{p}}\right)}_{nf}={\left(\widehat{\rho {C}_{p}}\right)}_{f}\left[\left(1-\phi \right)+\frac{\phi {\left(\widehat{\rho {C}_{p}}\right)}_{p}}{{\left(\widehat{\rho {C}_{p}}\right)}_{f}}\right],$$

3 $${\widehat{\mu }}_{nf}={\left(1-34.87{\left(\frac{{\stackrel{\smile}{d}}_{particle}}{{\stackrel{\smile}{d}}_{fluid}}\right)}^{-0.3}{\phi }^{1.03}\right)}^{-1}{\widehat{\mu }}_{f},$$

4 $${\widehat{k}}_{nf}={\widehat{k}}_{f}\left(1+4.4R{e}_{b}^{0.4}\stackrel{0.66}{\mathrm{Pr}}{\left(\frac{\widehat{T}}{{\widehat{T}}_{freezing}}\right)}^{10}{\left(\frac{{\widehat{k}}_{p}}{{\widehat{k}}_{f}}\right)}^{0.03}{\phi }^{0.66}\right).$$

The Reynolds number due to the influence of particles brownian motion is denoted by $R{e}_{b}$ and given by the following formula:

5 $$R{e}_{b}({\widehat{\mu }}_{f})={\widehat{d}}_{p}{\widehat{\rho }}_{f}{\widehat{u}}_{b},$$

where,

6 $${\widehat{u}}_{b}=2\widehat{T}{\widehat{k}}_{b}(\pi {\widehat{d}}_{p}^{2}{\widehat{\mu }}_{f}).$$

In Eq. (6), ${\widehat{k}}_{b}$ represents Stefan Boltzmann coefficient and its value is $1.380648\times {10}^{-23} (J{K}^{-1})$. Further, diameter of the nanomaterial is ${\widehat{d}}_{p}$ and is given in the following way^20^:

7 $${\widehat{d}}_{f}=6{M}^{*}{\left({N}^{*}{\widehat{\rho }}_{f}\pi \right)}^{-1}.$$

In Eq. (7), ${M}^{*}$ and ${N}^{*}$ are molecular weight and Avogadro number, respectively. The value of molecular diameter ${\widehat{d}}_{f}$ is described in the following formula:

8 $${\widehat{d}}_{f}={\left(\frac{6\times 0.01801528}{998.62\times \left(6.022\times {10}^{23}\right)\times \pi }\right)}^\frac{1}{3}=3.85\times {10}^{-10}nm,$$

9 $${\left(\widehat{\rho {\beta }_{T}}\right)}_{nf}=\left(1-\phi \right){\left(\widehat{\rho \beta }\right)}_{f}+\phi {\left(\widehat{\rho \beta }\right)}_{p},$$

10 $${\widehat{\sigma }}_{nf}={\widehat{\sigma }}_{f}\left(1+\frac{3\left(\frac{{\widehat{\sigma }}_{p}}{{\widehat{\sigma }}_{f}}-1\right)\phi }{\left(\left(\frac{{\widehat{\sigma }}_{p}}{{\widehat{\sigma }}_{f}}+2\right)-\left(\frac{{\widehat{\sigma }}_{p}}{{\widehat{\sigma }}_{f}}-1\right)\phi \right)}\right).$$

To improve thermal performance of the nanofluid, the following thermophysical values for Al_2_O_3_ and H_2_O are used and given in Table 1^19^:

**Table 1:** Thermophysical characteristics T = 310 K^19^.

Properties	${\widehat{d}}_{p}(\mathrm{nm})$	$\widehat{\rho }(\mathrm{kg}/{\mathrm{m}}^{3})$	$\widehat{\beta } (1/k)$	${\widehat{c}}_{p}(\mathrm{J}/\mathrm{Kg K})$	${\widehat{\mu }}_{f} (\mathrm{kg}/\mathrm{ms})$	$\widehat{k }(\mathrm{W}/\mathrm{mk})$	$\widehat{\sigma } (\mathrm{S}/\mathrm{m})$
${\mathrm{H}}_{2}\mathrm{O}$	$0.385$	$993$	$36.2\times {10}^{5}$	$4178$	$695{\times 10}^{6}$	$0.628$	$0.005$
Al_2_O_3_	33	$3970$	$0.85\times {10}^{5}$	$765$	–	$40$	$0.05\times {10}^{6}$

### Constitutive relations and boundary conditions

In the view of above restrictions, the following are boundary layer equations representing 3D flow of the nanofluid in the presence of freezing temperature and molecular diameter:

11 $$\frac{\partial\stackrel{\smile}{u}}{\partial x}+\frac{\partial\stackrel{\smile}{v}}{\partial y}+\frac{\partial\stackrel{\smile}{w}}{\partial z}=0,$$

12 $${\widehat{\rho }}_{nf}\left(\stackrel{\smile}{u}\frac{\partial\stackrel{\smile}{u}}{\partial x}+\stackrel{\smile}{v}\frac{\partial\stackrel{\smile}{u}}{\partial y}+\stackrel{\smile}{w}\frac{\partial\stackrel{\smile}{u}}{\partial z}\right)-{\widehat{\mu }}_{nf}\frac{{\partial }^{2}\stackrel{\smile}{u}}{\partial {z}^{2}}-\widehat{g}{\left(\widehat{\rho {\beta }_{T}}\right)}_{nf}\left(\widehat{T}-{\widehat{T}}_{\infty }\right)+{\widehat{\sigma }}_{nf}{B}^{2}\widehat{u}=0,$$

13 $${\widehat{\rho }}_{nf}\left(\stackrel{\smile}{u}\frac{\partial\stackrel{\smile}{v}}{\partial x}+\stackrel{\smile}{v}\frac{\partial\stackrel{\smile}{v}}{\partial y}+\stackrel{\smile}{w}\frac{\partial\stackrel{\smile}{v}}{\partial z}\right)-{\widehat{\mu }}_{nf}\frac{{\partial }^{2}\stackrel{\smile}{v}}{\partial {z}^{2}}+{\widehat{\sigma }}_{nf}{B}^{2}\widehat{v}=0,$$

14 $${\widehat{\rho }}_{nf}\left(\stackrel{\smile}{u}\frac{\partial\stackrel{\smile}{w}}{\partial x}+\stackrel{\smile}{v}\frac{\partial\stackrel{\smile}{w}}{\partial y}+\stackrel{\smile}{w}\frac{\partial\stackrel{\smile}{w}}{\partial z}\right)-{\widehat{\mu }}_{nf}\frac{{\partial }^{2}\stackrel{\smile}{w}}{\partial {z}^{2}}=0,$$

15 $${\left(\widehat{\rho {C}_{p}}\right)}_{nf}\left(\stackrel{\smile}{u}\frac{\partial\stackrel{\smile}{T}}{\partial x}+\stackrel{\smile}{v}\frac{\partial\stackrel{\smile}{T}}{\partial y}+\stackrel{\smile}{w}\frac{\partial\stackrel{\smile}{T}}{\partial z}\right)-{\widehat{k}}_{nf}\frac{{\partial }^{2}\stackrel{\smile}{T}}{\partial {z}^{2}}-{\widehat{\mu }}_{nf}\left({\left(\frac{\partial\stackrel{\smile}{u}}{\partial z}\right)}^{2}+{\left(\frac{\partial\stackrel{\smile}{v}}{\partial z}\right)}^{2}\right)-{\widehat{\sigma }}_{nf}{B}^{2}\left({\stackrel{\smile}{u}}^{2}+{\stackrel{\smile}{v}}^{2}\right)+\frac{16{\sigma }^{*}{\widehat{T}}_{\infty }^{3}}{3{K}^{*}}\frac{{\partial }^{2}\stackrel{\smile}{T}}{\partial {z}^{2}}=0.$$

The appropriate flow conditions in the presence of convective surface are described in the following manner:

At the surface:

$$\stackrel{\smile}{u}={\stackrel{\smile}{u}}_{w}+{\sigma }_{v}^{*}\left(2-{\sigma }_{v}^{*}\right){\lambda }_{0}\frac{\partial\stackrel{\smile}{u}}{\partial z},\stackrel{\smile}{v}={\stackrel{\smile}{v}}_{w}+{\sigma }_{v}^{*}\left(2-{\sigma }_{v}^{*}\right){\lambda }_{0}\frac{\partial\stackrel{\smile}{v}}{\partial z},\stackrel{\smile}{w}=0, {\widehat{k}}_{nf}\frac{\partial\stackrel{\smile}{T}}{\partial z}={\stackrel{\smile}{h}}_{f}(\stackrel{\smile}{T}-{\stackrel{\smile}{T}}_{f}).$$

Far from the surface:

$$\stackrel{\smile}{u}\to 0,\stackrel{\smile}{v}\to 0,\stackrel{\smile}{T}\to {\stackrel{\smile}{T}}_{\infty }$$

where, stretching velocities are denoted by ${\stackrel{\smile}{u}}_{w}$ and ${\stackrel{\smile}{v}}_{w}$, the surface is heated due to thermal transfer coefficient ${\stackrel{\smile}{h}}_{f}$, velocity coefficient is ${\lambda }_{0}$. These are taken as variable kind and given by the following mathematical relations:

${\lambda }_{0}={\stackrel{\smile}{\lambda }}_{0}{\left(x+y\right)}^{0.5(1-n)}$ and ${\stackrel{\smile}{h}}_{f}=\stackrel{\smile}{c}{\left({x}^{1-n}\right)}^{-0.5}$, respectively. The terms ${\stackrel{\smile}{\lambda }}_{0}$ and $\stackrel{\smile}{c}$ are constants.

### Invertible equations

In order to continue the nondimensionalization process, the following invertible relations are introduced for the momentum and energy equations:

16 $$\left.\begin{array}{c}\stackrel{\smile}{u}=\stackrel{\smile}{a}{\left(x+y\right)}^{n}{F}^{^{\prime}},\stackrel{\smile}{v}=\stackrel{\smile}{a}{\left(x+y\right)}^{n}{G}^{^{\prime}}\\\stackrel{\smile}{w}=-{\left(\stackrel{\smile}{a}{\nu }_{f}\right)}^{0.5}{\left(x+y\right)}^{0.5\left(n-1\right)}\left[0.5\left(n+1\right)\left(F+G\right)+0.5\left(n-1\right)\eta \left({F}^{^{\prime}}+{G}^{^{\prime}}\right)\right]\\\stackrel{\smile}{T}={\stackrel{\smile}{T}}_{\infty }+\left({\stackrel{\smile}{T}}_{f}-{\stackrel{\smile}{T}}_{\infty }\right)\beta , \eta ={\left(\frac{a}{{\nu }_{f}}\right)}^{0.5}{\left(x+y\right)}^{0.5(n-1)}z\end{array}\right\}.$$

### Al_2_O_3_-H_2_O model

The nondimensionalization process is carried out in the view of above-described dimensional model and supporting conditions at the surface and away from it. Finally, the following model is attained by incorporating the effective nanofluid correlations:

17 $$\frac{F{^{\prime}}{^{\prime}}{^{\prime}}}{1-34.87{\left(\frac{{d}_{particle}}{{d}_{fluid}}\right)}^{-0.3}{\phi }^{1.03}}+\frac{0.5\left(n+1\right)\left(F+G\right){F}^{{^{\prime}}{^{\prime}}}-n({F}^{^{\prime}}+G{^{\prime}})F{^{\prime}}}{{\left(\left(1-\phi \right)+\frac{\phi {\widehat{\rho }}_{p}}{{\widehat{\rho }}_{f}}\right)}^{-1}}+\frac{\beta \lambda }{{\left(\left(1-\phi \right)+\frac{\phi {\left(\widehat{\rho \beta }\right)}_{p}}{{\left(\widehat{\rho \beta }\right)}_{f}}\right)}^{-1}}-\frac{{M}^{2}{F}^{^{\prime}}}{{\left(1+\frac{3\left(\frac{{\widehat{\sigma }}_{p}}{{\widehat{\sigma }}_{f}}-1\right)\phi }{\left(\left(\frac{{\widehat{\sigma }}_{p}}{{\widehat{\sigma }}_{f}}+2\right)-\left(\frac{{\widehat{\sigma }}_{p}}{{\widehat{\sigma }}_{f}}-1\right)\phi \right)}\right)}^{-1}}=0,$$

18 $$\frac{G{^{\prime}}{^{\prime}}{^{\prime}}}{1-34.87{\left(\frac{{d}_{particle}}{{d}_{fluid}}\right)}^{-0.3}{\phi }^{1.03}}+\frac{0.5\left(n+1\right)\left(F+G\right){G}^{{^{\prime}}{^{\prime}}}-n({F}^{^{\prime}}+G{^{\prime}})G{^{\prime}}}{{\left(\left(1-\phi \right)+\frac{\phi {\widehat{\rho }}_{p}}{{\widehat{\rho }}_{f}}\right)}^{-1}}-\frac{{M}^{2}{G}^{^{\prime}}}{{\left(1+\frac{3\left(\frac{{\widehat{\sigma }}_{p}}{{\widehat{\sigma }}_{f}}-1\right)\phi }{\left(\left(\frac{{\widehat{\sigma }}_{p}}{{\widehat{\sigma }}_{f}}+2\right)-\left(\frac{{\widehat{\sigma }}_{p}}{{\widehat{\sigma }}_{f}}-1\right)\phi \right)}\right)}^{-1}}=0,$$

19 $${\widehat{k}}_{nf}\left(\frac{4Rd+3}{3Pr}\right){\beta }^{{^{\prime}}{^{\prime}}}+\frac{0.5\left(n+1\right)\left(F+G\right)\beta {^{\prime}}}{{\left(\left(1-\phi \right)+\frac{\phi {\left(\widehat{\rho {C}_{p}}\right)}_{p}}{{\left(\widehat{\rho {C}_{p}}\right)}_{f}}\right)}^{-1}}+\frac{Ec({F}^{{^{\prime}}{^{\prime}}2}+{G}^{{^{\prime}}{^{\prime}}2})}{1-34.87{\left(\frac{{d}_{particle}}{{d}_{fluid}}\right)}^{-0.3}{\phi }^{1.03}}+\frac{{EcM}^{2}({F}^{{^{\prime}}2}+{G}^{{^{\prime}}2})}{{\left(1+\frac{3\left(\frac{{\widehat{\sigma }}_{p}}{{\widehat{\sigma }}_{f}}-1\right)\phi }{\left(\left(\frac{{\widehat{\sigma }}_{p}}{{\widehat{\sigma }}_{f}}+2\right)-\left(\frac{{\widehat{\sigma }}_{p}}{{\widehat{\sigma }}_{f}}-1\right)\phi \right)}\right)}^{-1}}=0.$$

The related nondimensional version of the flow conditions is as under:

$${F}^{^{\prime}}=1+\stackrel{\smile}{S}{F}^{{^{\prime}}{^{\prime}}}, {G}^{^{\prime}}=c+\stackrel{\smile}{S}{G}^{{^{\prime}}{^{\prime}}}, F=0, G=0,\frac{{\widehat{k}}_{nf}}{{\widehat{k}}_{f}}\beta {^{\prime}}={B}_{i}(\beta -1)$$ at $$\eta =0$$

$${F}^{^{\prime}}\to 0,{G}^{^{\prime}}\to 0,\beta \to 0$$ as $$\eta \to \infty$$

Where, $F$, $\beta$ and their derivatives depend on dimensionless variable $\eta$. Further, the parameters embedded in the model are magnetic number (${M}^{2}=\frac{{\stackrel{\smile}{\sigma }}_{f}{B}_{0}^{2}}{\stackrel{\smile}{a}{\stackrel{\smile}{\rho }}_{f}}$), mixed convection number ($\lambda =\frac{{G}_{rx}}{R{e}_{x}^{2}}$), Grashof parameter (${G}_{rx}=\stackrel{\smile}{g}{\stackrel{\smile}{\beta }}_{Tf}\left({\stackrel{\smile}{T}}_{f}-{\stackrel{\smile}{T}}_{\infty }\right){\left(x+y\right)}^{3}{\nu }_{f}^{-1}$), thermal radiation number ($Rd=\frac{16{\sigma }^{*}{\stackrel{\smile}{T}}_{\infty }^{3}}{3{\stackrel{\smile}{k}}_{f}{k}^{*}}$), Prandtl number ($Pr=\frac{{\left(\stackrel{\smile}{\mu }{C}_{p}\right)}_{f}}{{\stackrel{\smile}{k}}_{f}}$), Eckert parameter ($Ec=\frac{{\stackrel{\smile}{u}}_{w}^{2}}{{C}_{pf}({\stackrel{\smile}{T}}_{f}-{\stackrel{\smile}{T}}_{\infty })}$), stretching ratio number ($c=\frac{\stackrel{\smile}{b}}{\stackrel{\smile}{a}}$), the velocity slip ($S=\frac{2-{\sigma }_{v}}{{\sigma }_{v}}{\lambda }_{0}{\left(\frac{\stackrel{\smile}{a}}{{\nu }_{f}}\right)}^{0.5}$) and Biot parameter (${B}_{i}=\frac{{c}^{*}}{{\stackrel{\smile}{k}}_{f}}{\left(\frac{{\nu }_{f}}{\stackrel{\smile}{a}}\right)}^{0.5}$). These parameter values are given in Table 2 for the velocity and temperature regimes:

While, these parameters are varied from $0.0$ to $1.0$ for the shear stresses and local Nusselt number.

The behavior of shear stresses and local thermal transport rate at the surface is described by the following equations:

$${C}_{fx}=\frac{{\stackrel{\smile}{\mu }}_{nf}}{{\stackrel{\smile}{\rho }}_{f}{\stackrel{\smile}{u}}_{w}^{2}}{\left(\frac{\partial\stackrel{\smile}{u}}{\partial z}+\frac{\partial\stackrel{\smile}{w}}{\partial x}\right)}_{z=0}$$

$${C}_{fy}=\frac{{\stackrel{\smile}{\mu }}_{nf}}{{\stackrel{\smile}{\rho }}_{f}{\stackrel{\smile}{u}}_{w}^{2}}{\left(\frac{\partial\stackrel{\smile}{v}}{\partial z}+\frac{\partial\stackrel{\smile}{w}}{\partial y}\right)}_{z=0}$$

$$Nu=\frac{x+y}{{\stackrel{\smile}{k}}_{f}\left({\stackrel{\smile}{T}}_{f}-{\stackrel{\smile}{T}}_{\infty }\right)}(-{\stackrel{\smile}{k}}_{nf}{\left(\frac{\partial\stackrel{\smile}{T}}{\partial z}\right)}_{z=0}+{\stackrel{\smile}{qr}}_{w})$$

After using the values of derivatives, the following version is obtained:

$${C}_{fx}R{e}_{x}^{0.5}=\frac{F{^{\prime}}{^{\prime}}(0)}{1-34.87{\left(\frac{{d}_{particle}}{{d}_{fluid}}\right)}^{-0.3}{\phi }^{1.03}}$$

$${C}_{fy}R{e}_{v}^{0.5}=\frac{G{^{\prime}}{^{\prime}}(0)}{1-34.87{\left(\frac{{d}_{particle}}{{d}_{fluid}}\right)}^{-0.3}{\phi }^{1.03}}$$

$$N{u}_{x}R{e}_{x}^{0.5}=-\frac{\left(1+4.4R{e}_{b}^{0.4}\stackrel{0.66}{\mathrm{Pr}}{\left(\frac{\widehat{T}}{{\widehat{T}}_{freezing}}\right)}^{10}{\left(\frac{{\widehat{k}}_{p}}{{\widehat{k}}_{f}}\right)}^{0.03}{\phi }^{0.66}\right)}{{\widehat{k}}_{f}}\left(1+\frac{4}{3}Rd\right){\beta }^{\mathrm{^{\prime}}}\left(0\right).$$

In which, $R{e}_{x}^{0.5}=\frac{{\stackrel{\smile}{u}}_{w}\left(x+y\right)}{{\nu }_{f}}$ and $R{e}_{y}^{0.5}=\frac{{\stackrel{\smile}{v}}_{w}\left(x+y\right)}{{\nu }_{f}}$ and these are known as local Reynolds number.

## Mathematical analysis of Al_2_O_3_-H_2_O model

It is investigated that the numerical techniques that are adopted by the researchers and engineers to tackle the flow model that usually comprises the set of ordinary differential equations, among these methods RK scheme along with shooting algorithm is very suitable and it has high accuracy. The solution of the model in this regard based on the transformation of higher order system of ODEs into the system of first order ODEs. For the particular model, the following relations was developed to transform it into to the desired system:

20 $$F\left(\eta \right)={\mathcal{A}}_{1}^{*},{F}^{\mathrm{^{\prime}}}\left(\eta \right)={\mathcal{A}}_{2}^{*},{F}^{\mathrm{^{\prime}}\mathrm{^{\prime}}}\left(\eta \right)={\mathcal{A}}_{3}^{*},{F}^{\mathrm{^{\prime}}\mathrm{^{\prime}}\mathrm{^{\prime}}}\left(\eta \right)={{\mathcal{A}}^{*}}_{3}^{\mathrm{^{\prime}}},G\left(\eta \right)={\mathcal{B}}_{4}^{*},{G}^{\mathrm{^{\prime}}}\left(\eta \right)={\mathcal{B}}_{5}^{*}, {G}^{\mathrm{^{\prime}}\mathrm{^{\prime}}}\left(\eta \right)={\mathcal{B}}_{6}^{*}, {G\mathrm{^{\prime}}}^{\mathrm{^{\prime}}\mathrm{^{\prime}}}\left(\eta \right)={{\mathcal{B}}^{*}}_{6}^{\mathrm{^{\prime}}},\beta \left(\eta \right)={\mathcal{C}}_{7}^{*}, {\beta }^{\mathrm{^{\prime}}}\left(\eta \right)={\mathcal{C}}_{8}^{*},{\beta }^{\mathrm{^{\prime}}\mathrm{^{\prime}}}\left(\eta \right)={{\mathcal{C}}^{*}}_{9}^{\mathrm{^{\prime}}},\dots .$$

By implementing these transformations, the following system is obtained:

21 $$\frac{{{\mathcal{A}}^{*}}_{3}^{^{\prime}}}{1-34.87{\left(\frac{{d}_{particle}}{{d}_{fluid}}\right)}^{-0.3}{\phi }^{1.03}}+\frac{0.5\left(n+1\right)\left({\mathcal{A}}_{1}^{*}+{\mathcal{B}}_{4}^{*}\right){\mathcal{A}}_{3}^{*}-n({\mathcal{A}}_{2}^{*}+{\mathcal{B}}_{5}^{*}){\mathcal{A}}_{2}^{*}}{{\left(\left(1-\phi \right)+\frac{\phi {\widehat{\rho }}_{p}}{{\widehat{\rho }}_{f}}\right)}^{-1}}+\frac{{\mathcal{C}}_{7}^{*}\lambda }{{\left(\left(1-\phi \right)+\frac{\phi {\left(\widehat{\rho \beta }\right)}_{p}}{{\left(\widehat{\rho \beta }\right)}_{f}}\right)}^{-1}}-\frac{{M}^{2}{\mathcal{A}}_{2}^{*}}{{\left(1+\frac{3\left(\frac{{\widehat{\sigma }}_{p}}{{\widehat{\sigma }}_{f}}-1\right)\phi }{\left(\left(\frac{{\widehat{\sigma }}_{p}}{{\widehat{\sigma }}_{f}}+2\right)-\left(\frac{{\widehat{\sigma }}_{p}}{{\widehat{\sigma }}_{f}}-1\right)\phi \right)}\right)}^{-1}}=0,$$

22 $$\frac{{{\mathcal{B}}^{*}}_{6}^{^{\prime}}}{1-34.87{\left(\frac{{d}_{particle}}{{d}_{fluid}}\right)}^{-0.3}{\phi }^{1.03}}+\frac{0.5\left(n+1\right)\left({\mathcal{A}}_{1}^{*}+{\mathcal{B}}_{4}^{*}\right){\mathcal{B}}_{6}^{*}-n({\mathcal{A}}_{2}^{*}+{\mathcal{B}}_{5}^{*}){\mathcal{B}}_{5}^{*}}{{\left(\left(1-\phi \right)+\frac{\phi {\widehat{\rho }}_{p}}{{\widehat{\rho }}_{f}}\right)}^{-1}}-\frac{{M}^{2}{\mathcal{B}}_{5}^{*}}{{\left(1+\frac{3\left(\frac{{\widehat{\sigma }}_{p}}{{\widehat{\sigma }}_{f}}-1\right)\phi }{\left(\left(\frac{{\widehat{\sigma }}_{p}}{{\widehat{\sigma }}_{f}}+2\right)-\left(\frac{{\widehat{\sigma }}_{p}}{{\widehat{\sigma }}_{f}}-1\right)\phi \right)}\right)}^{-1}}=0,$$

23 $${\widehat{k}}_{nf}\left(\frac{4Rd+3}{3Pr}\right){{\mathcal{C}}^{*}}_{9}^{^{\prime}}+\frac{0.5\left(n+1\right)\left({\mathcal{A}}_{1}^{*}+{\mathcal{B}}_{4}^{*}\right){\mathcal{C}}_{8}^{*}}{{\left(\left(1-\phi \right)+\frac{\phi {\left(\widehat{\rho {C}_{p}}\right)}_{p}}{{\left(\widehat{\rho {C}_{p}}\right)}_{f}}\right)}^{-1}}+\frac{Ec({{\mathcal{A}}_{3}^{*}}^{2}+{{\mathcal{B}}_{6}^{*}}^{2})}{1-34.87{\left(\frac{{d}_{particle}}{{d}_{fluid}}\right)}^{-0.3}{\phi }^{1.03}}+\frac{{EcM}^{2}({{\mathcal{A}}_{2}^{*}}^{2}+{{\mathcal{B}}_{5}^{*}}^{2})}{{\left(1+\frac{3\left(\frac{{\widehat{\sigma }}_{p}}{{\widehat{\sigma }}_{f}}-1\right)\phi }{\left(\left(\frac{{\widehat{\sigma }}_{p}}{{\widehat{\sigma }}_{f}}+2\right)-\left(\frac{{\widehat{\sigma }}_{p}}{{\widehat{\sigma }}_{f}}-1\right)\phi \right)}\right)}^{-1}}=0.$$

The reduced nanofluid model is then treated by using Mathematica 10.0 version and plotted the results for the flow regimes against multiple flow quantities over the region of interest.

## Graphical results with discussion

### Al_2_O_3_-H_2_O velocity against M and S

**Figure 2:**
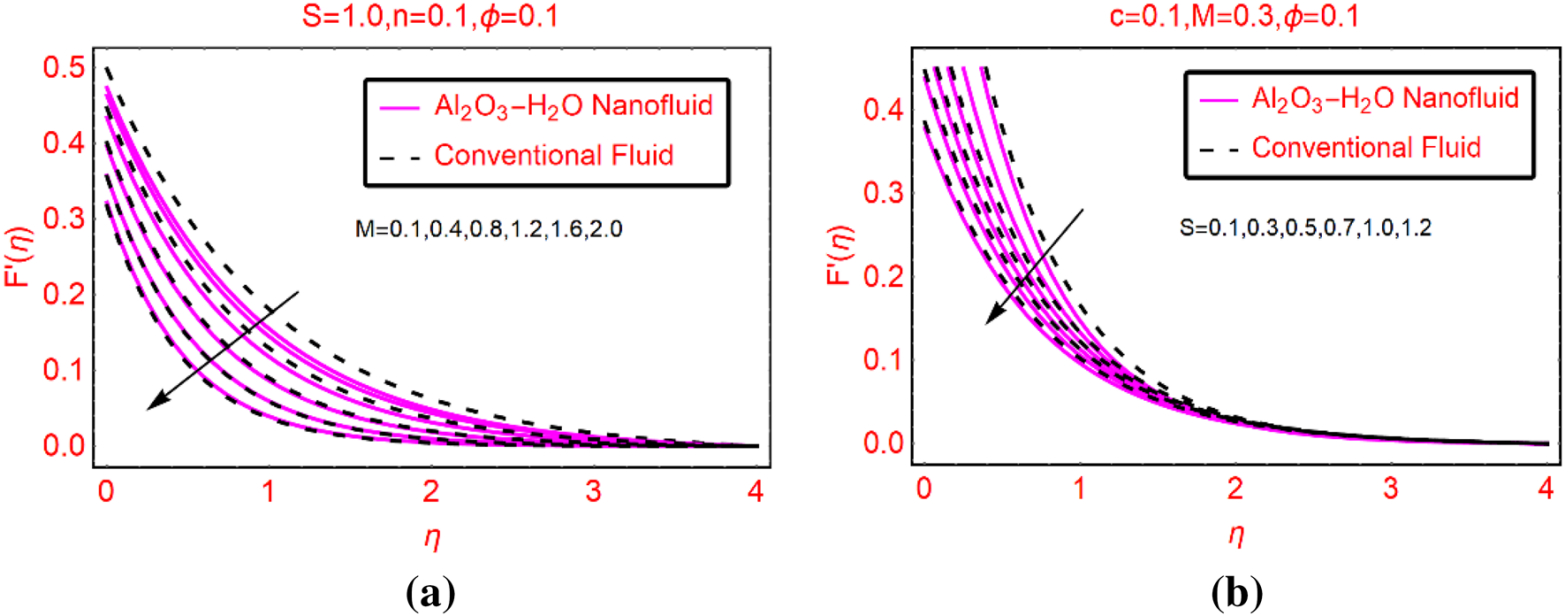
$$F{^{\prime}}(\eta )$$ against **(a)** M and **(b)** S.

The analysis of Lorentz forces on the fluid motion is significant from industrial point of view. This type of physical situation is broadly used in order to purify the different industrial products. The results are displayed for conventional versus nanofluid over the feasible region. Figure 2a elaborating the behaviour of the fluid motion against magnetic parameter M. From this, it is cleared that the fluid moves slowly by increasing the strength of magnetic field. Physical theme behind this fluid behavior is that the Lorentz forces resists the fluid motion^21^. Further, for Al_2_O_3_-H_2_O nanofluid, the motion decreases abruptly comparative to the conventional liquid. Physically, the conventional liquid is less dense than the nanofluid and its particles moves freely compared the nanofluid. Therefore, the conventional liquid moves abruptly than the nanofluid. The influences of velocity slip on axial motion of the fluids are elucidated in Fig. 2b. The prominent changes in the fluid motion are observed near the slippery surface and asymptotic behavior is noted beyond $\eta >0$.

### Al_2_O_3_-H_2_O velocity against n and c

**Figure 3:**
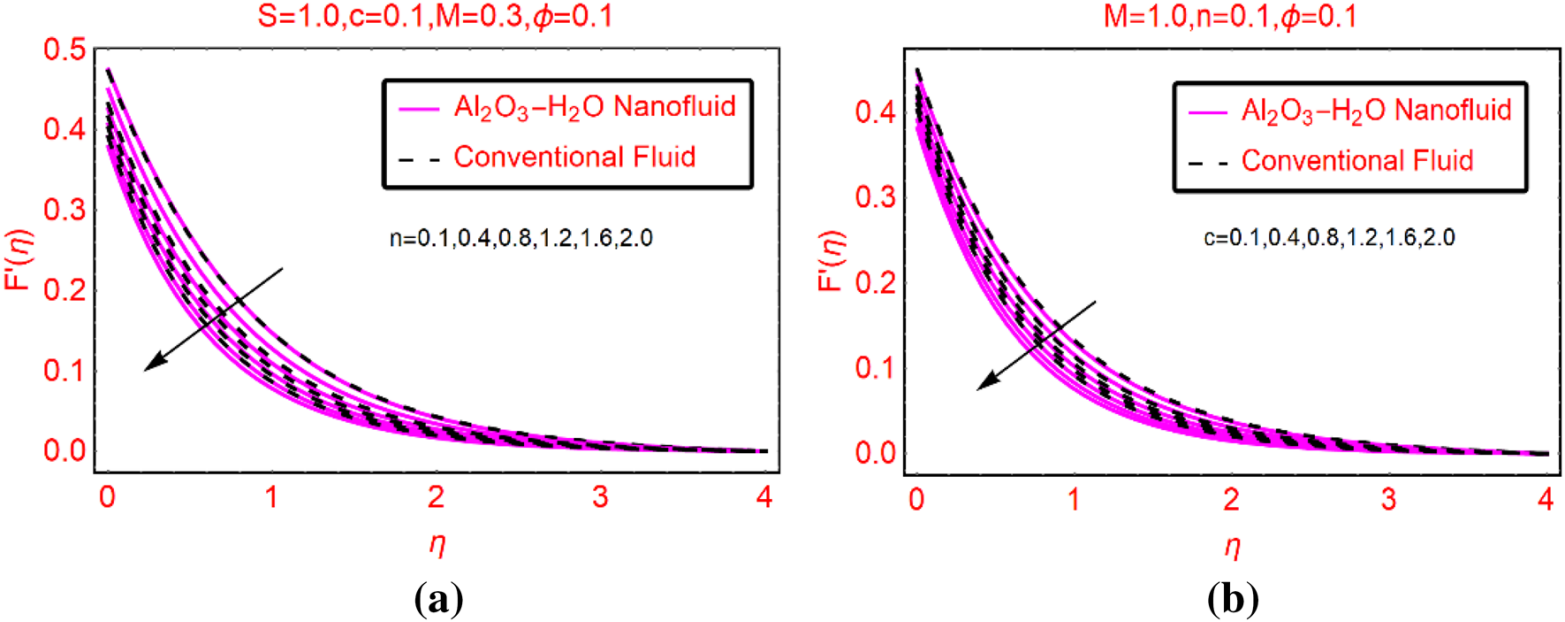
$$F{^{\prime}}(\eta )$$ against **(a)** n and **(b)** c.

The alterations in axial motion of the fluid against n and stretching number c are pictured in Fig. 3a,b, respectively. From the plotted results, it is investigated that the fluid motion declines in both situations. However, for stretchable surface these effects are quite slow. Moreover, abrupt decreasing behavior of Al_2_O_3_-H_2_O is observed due to their thermophysical values.

### Al_2_O_3_-H_2_O transverse velocity against M and S

**Figure 4:**
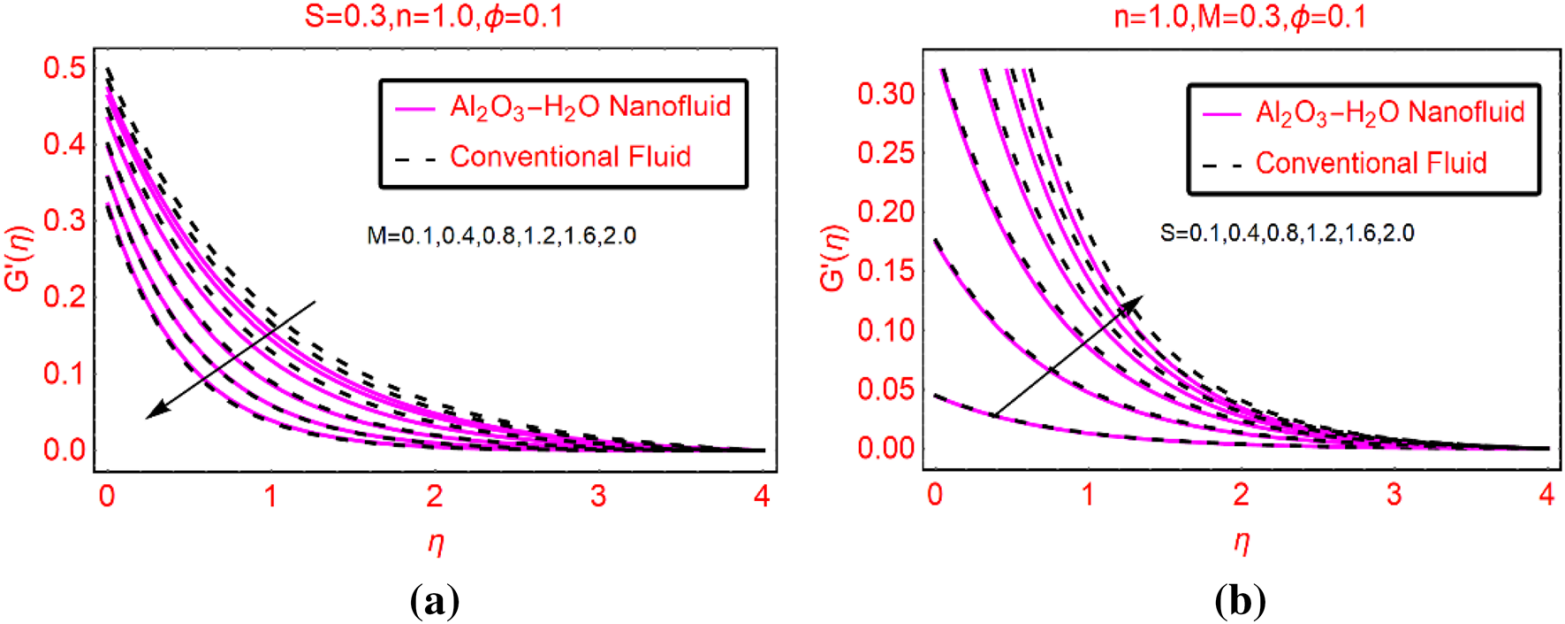
$$G{^{\prime}}(\eta )$$ against **(a)** M and **(b)** S.

The transverse component of the velocity due to altering M and S are described in Fig. 4a,b, respectively. It is examined that the fluid motion drops transversely due to stronger imposed magnetic field. The maximum decrement can be observed near the surface due to the prominent influence of the magnetic field which opposes the motion and it gradually becomes slow down far from the surface. The fluid motion becomes stable far from the surface and it showing asymptotic behavior. These influences are sketched in Fig. 4a. On the other hand, transverse velocity against the varying slippery surface is decorated in Fig. 4b. The velocity boosted significantly against more slippery surface. Physically, the fluid particles move rapidly due to the velocity slip parameter and significantly affects the transverse motion near the surface.

### Al_2_O_3_-H_2_O thermal behaviour against Ec and B_i_

**Figure 5:**
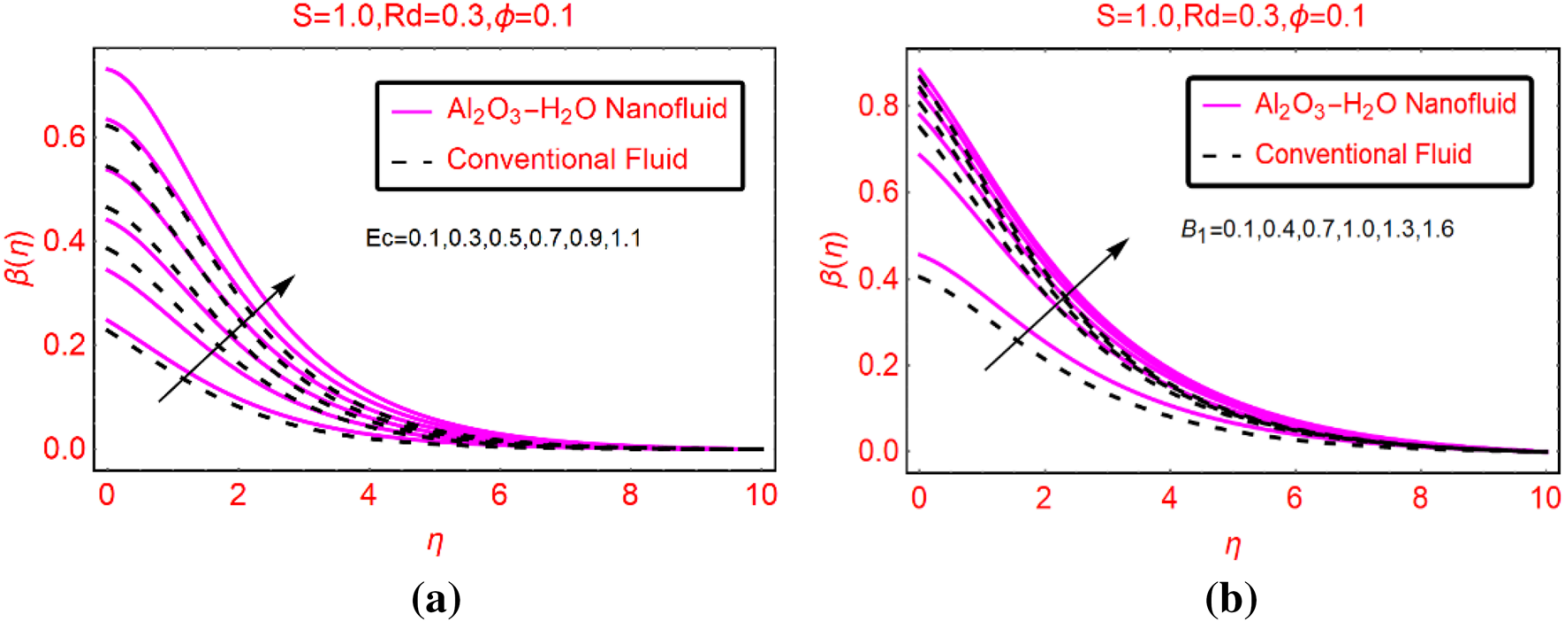
$\beta (\eta )$ against (a) Ec and (b) B_i_.

The viscous dissipation and convective heating are significant physical phenomenon that playing vital role in the fluid thermal behavior. Therefore, the fluid thermal behavior $\beta (\eta )$ by varying Ec and B_i_ parameters are elaborated in Fig. 5a,b, respectively. From Fig. 5a, it is noted that the fluid temperature enhances rapidly due to more dissipative fluid. For Al_2_O_3_-H_2_O nanofluid, the temperature rises rapidly than the conventional liquid. Primarily, thermal conductivity of the nanofluid is playing significant role for thermal enhancement in the nanofluid. Therefore, the nanofluid has high thermal transport capability than the regular liquid. The temperature due to convectively heated surface is elaborated in Fig. 5b. Physically, convectively heated sheet provides an extra heat to the fluid particles in the presence of viscous dissipation. It boosts the fluid temperature effectively near the surface. Physically, maximum rise in the temperature near the sheet vicinity is due to the stronger convective condition effects. Initially, the heat transfer occurs due to the direct contact of the fluid particles adjacent to the surface (heat conduction) and then these particles move from higher to lower temperature region by following the heat convection mechanism. Meanwhile, imposed thermal radiations additionally provide the energy to the nanofluid which ultimately increase the nanofluid temperature.

### Al_2_O_3_-H_2_O thermal behaviour against Rd and M

**Figure 6:**
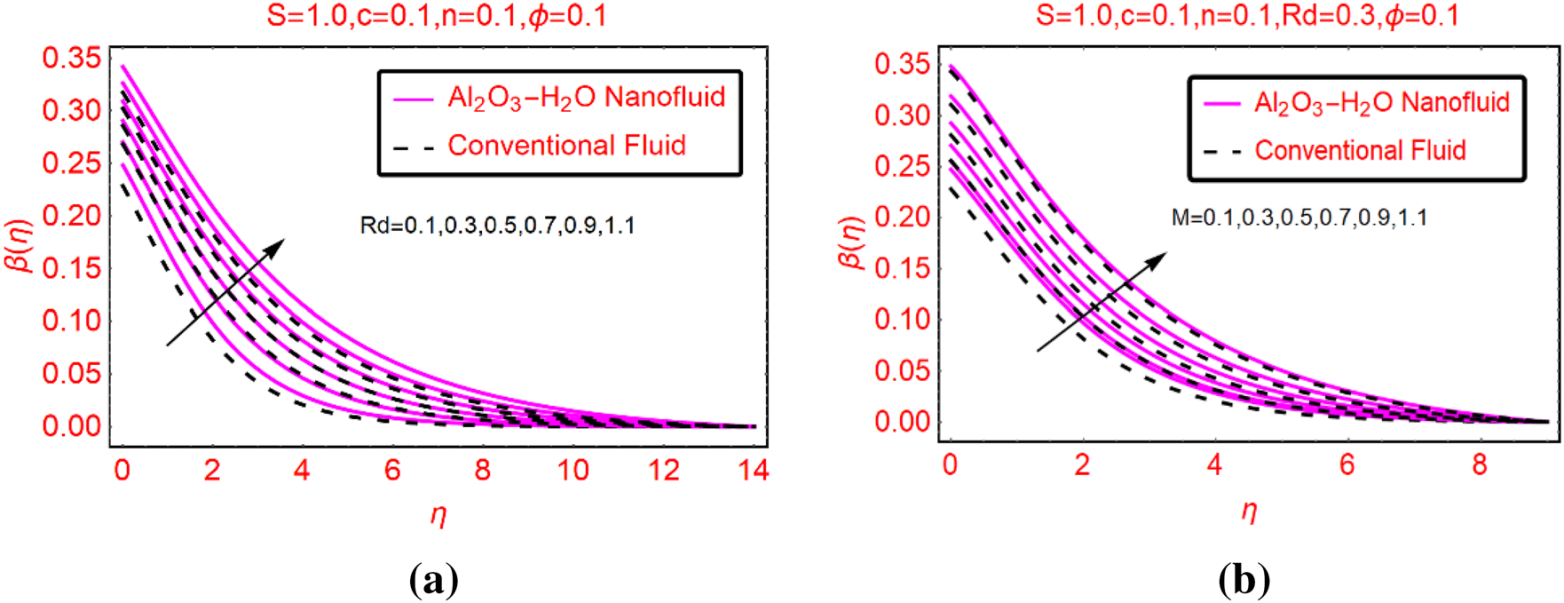
$\beta (\eta )$ against **(a)** Rd and **(b)** M.

Thermal radiation and magnetic field are significant physical aspects which playing important role in the fluid temperature. These effects are elucidated in Fig. 6a,b, respectively. It is concluded that the fluid temperature boosts in the existence of thermal radiation and magnetic field. Physically, the fluid gains extra energy due to imposed thermal radiation which ultimately boosts the temperature. The low increment in the regular liquid is examined because its thermal transport characteristics are not good than nanofluid. Further, the impacts of these parameters on the temperature are almost vanishes far from the surface.

### Quantities of engineering interest

**Figure 7:**
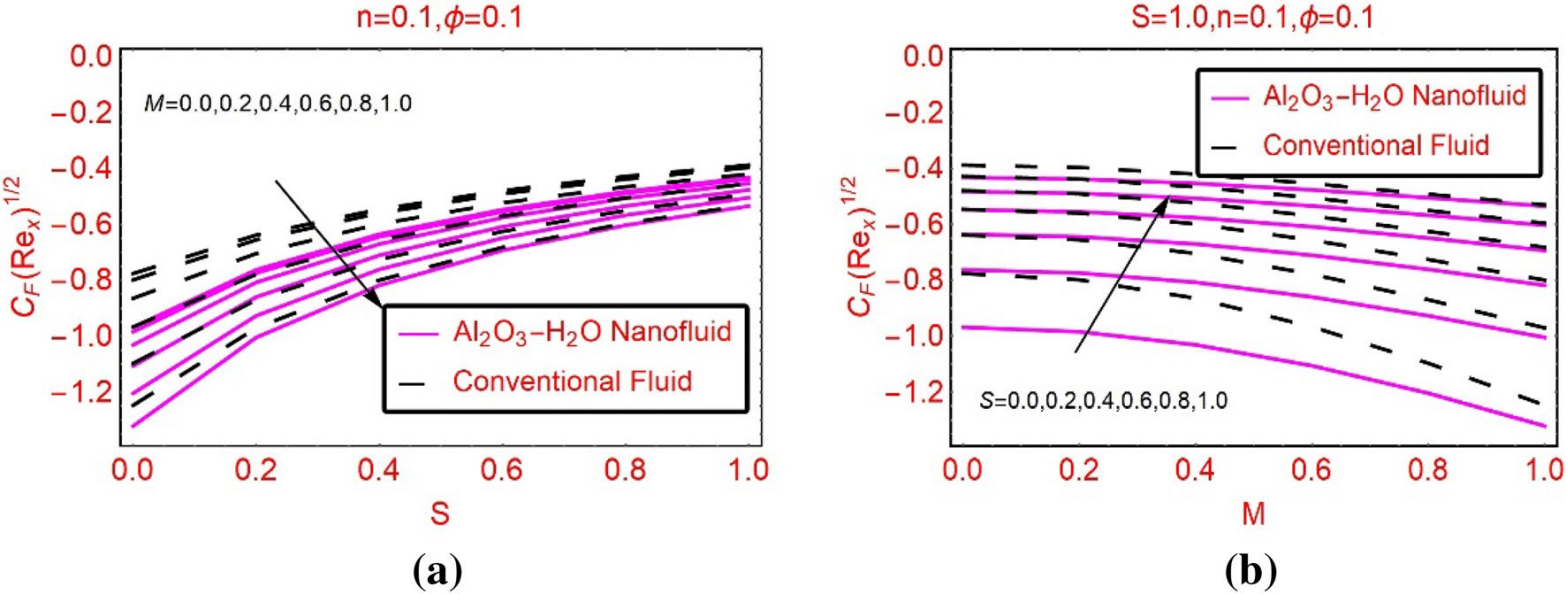
$${C}_{fx}R{e}_{x}^{0.5}$$ against **(a)** M and **(b)** S.

**Figure 8:**
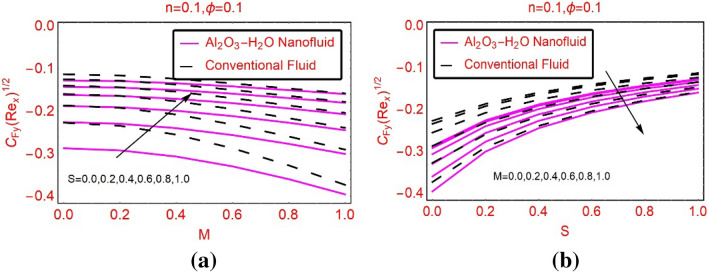
$${C}_{fy}R{e}_{x}^{0.5}$$ against **(a)** S and **(b)** M.

This subsection highlights the trends in the shears stresses and local heat transport rate against the parameters embedded in the model. Figure 7a,b highlighting the behaviour of the shear stresses on the surface against varying M and S, respectively. It is observed that the shear stresses drop for stronger magnetic field and maximum decrement is noted for the nanofluid. Physically, the nanofluid is denser due to their effective density and hence the shear stresses decline abruptly. On the other hand, the velocity slip effects favor it and these effects are portrayed in Fig. 7b. The transverse shear stresses against S and M are pictured in Fig. 8a,b, respectively. It is noticed that the transverse stresses also declines but, this decrement is very rapid than the axial shear stresses.

**Figure 9:**
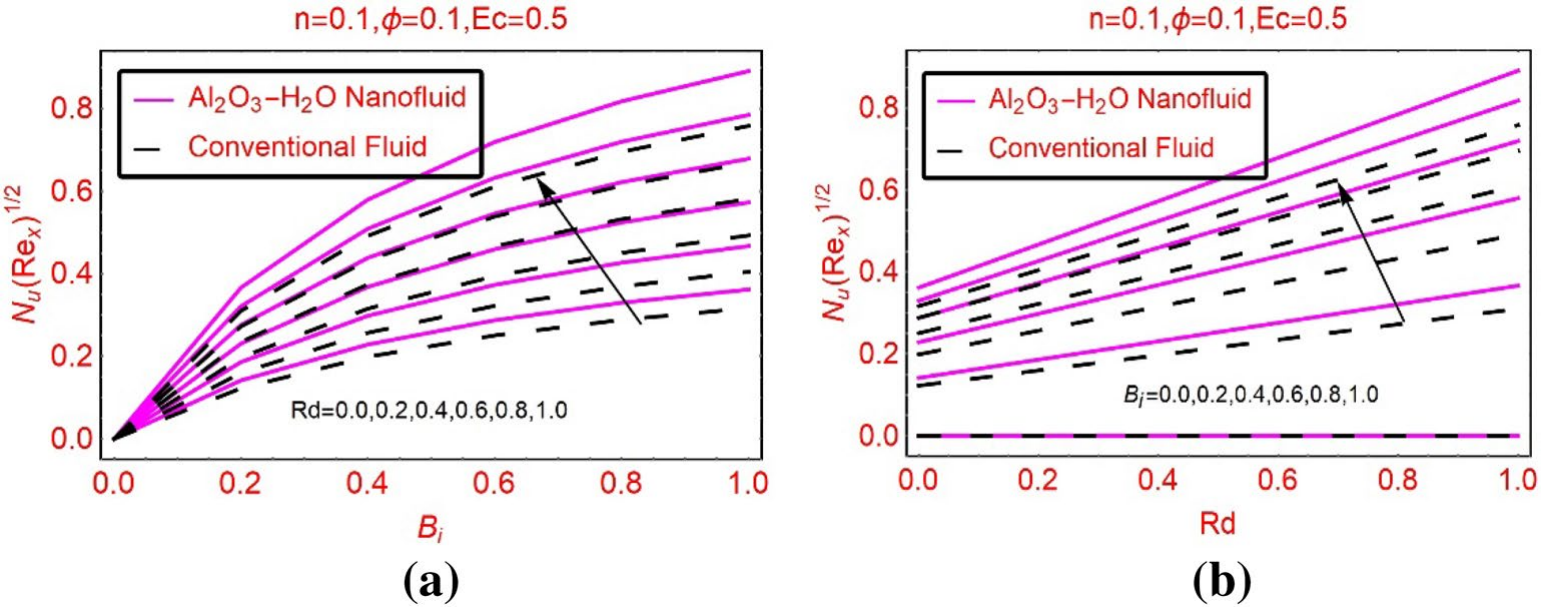
$NuR{e}_{x}^{0.5}$ against **(a)** Rd and **(b)** B_i_.

The local thermal performance rate in the nanofluid as well as regular liquid changes significantly by altering the flow quantities. Therefore, Fig. 9 is plotted to study the behavior of local Nusselt number against thermal radiation and Biot number. From concerned Fig. 9a,b, it is examined that thermal radiations and convectively heated surface playing significant role regarding the local thermal performance rate in the nanofluid as well as regular liquid. From this, it seems that the nanofluid have high thermal performance rate and is useful for industrial applications. Due to convectively heated surface, the rate of local heat transport is maximum because heated surface transfers the heat to the fluid particles which boosts the local heat transport rate. These trends are elaborated in Fig. 9b.

### Streamlines trends

**Figure 10:**
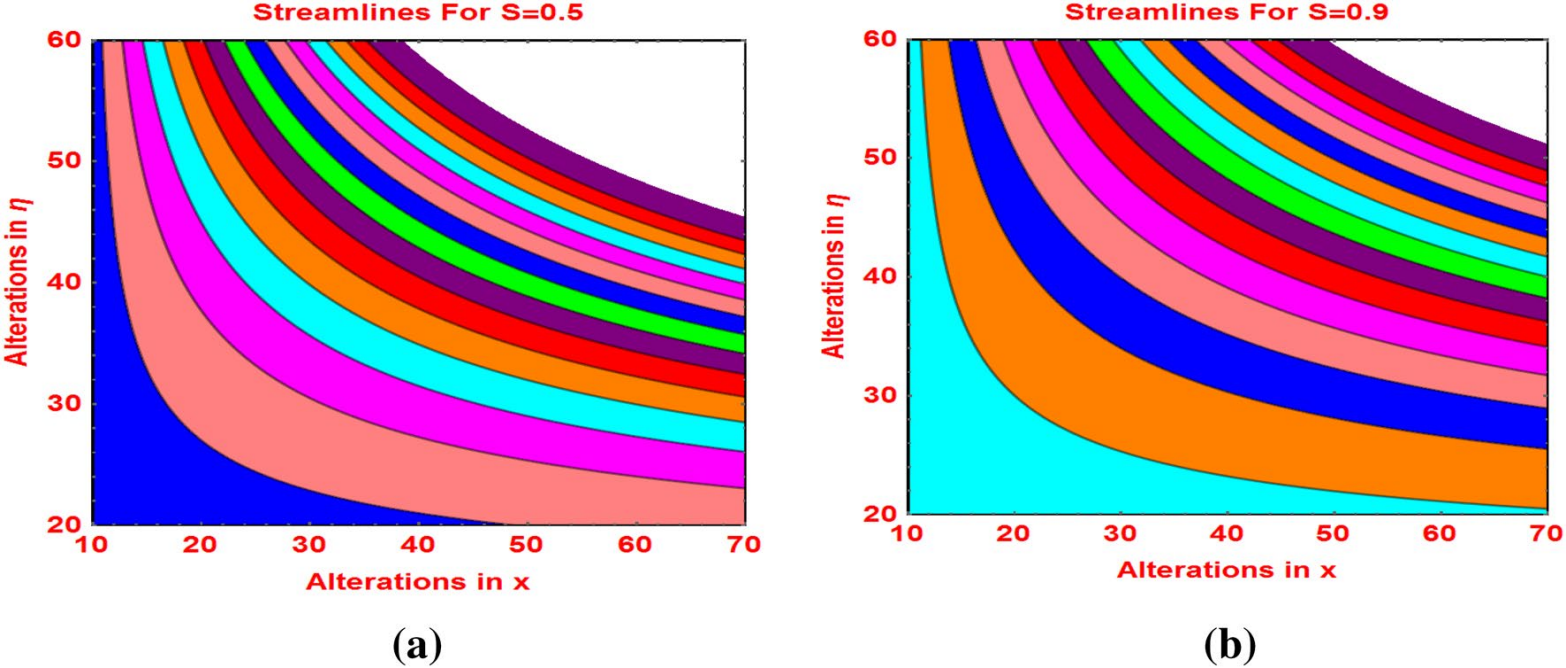
Streamlines against **(a)** $S=0.5$ and **(b)**$S=0.9.$

**Figure 11:**
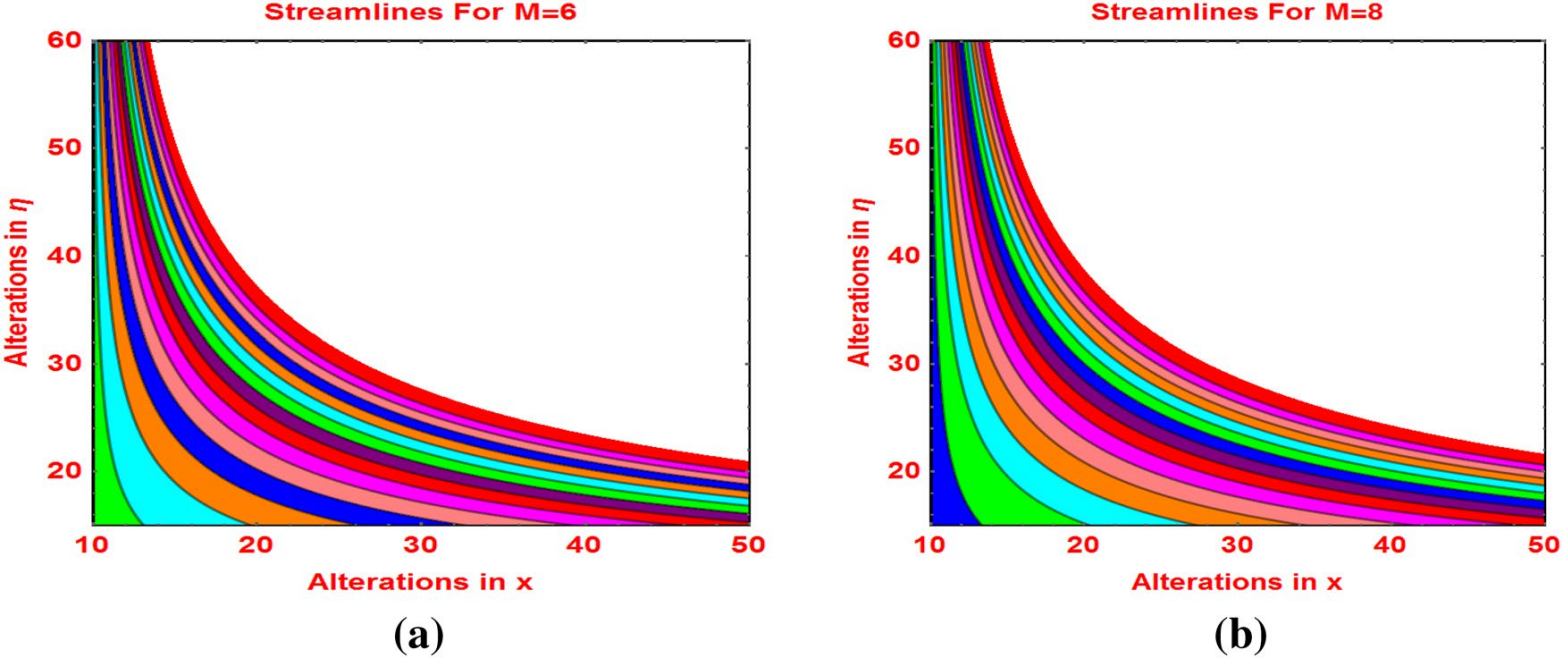
Streamlines against **(a)** M = 6 and **(b)** M = 8.

The trends in the flow streamlines against the velocity slip parameter and magnetic field are sketched in Figs. 10a,b, 11a,b, respectively. The streamlines are become flatten against the velocity slip and more bended behavior is noticed for stronger effects of the Lorentz forces and these are elucidated in Fig. 11.

### Authenticity of the analysis

The comparison of present study with previously reported science literature is organized in this section. The under-consideration model can be reduced into the conventional liquid by setting $\phi =0$ and to meet the requirements of previously reported model, we also took $M=S=\lambda =0$. The numerical computation is than carried out by setting ${\eta }_{\infty }=11.0$. From the computation, it is examined that the present results and implemented numerical technique is accurate with the results reported in^21^. This provides the reliability of the study. The computed results are given in Table 3.

**Table 2:** Ranges of the parameters for the velocity and temperature fields.

Name of parameter	Range of variation	Name of parameter	Range of variation
$M$	0.1–2.0	$Ec$	0.1–1.1
$n$	0.1–2.0	${B}_{i}$	0.1–1.6
$c$	0.1–2.0	$Rd$	0.1–1.1
$S$	0.1–1.2	–	–

**Table 3:** Comparison of present study with previously reported studies.

$c$	$F{^{\prime}}{^{\prime}}(0)$ current results	Khan et al.^21^	${G}^{{^{\prime}}{^{\prime}}}\left(0\right)$ current results	Khan et al.^22^
$0.0$	$-1.0$	$-1.0$	$0.0$	$0.0$
$0.5$	$-1.22474$	$-1.22474$	$-0.612373$	$-0.61237$
$1.0$	$-1.41421$	$-1.41421$	$-1.41421$	$-1.41421$

### Findings

Analysis of the dynamics of Al_2_O_3_-H_2_O over a vertically convectively heated plate is taken by considering the influences of imposed Lorentz forces, thermal radiations and viscous dissipation. The problem is modeled by utilizing nanofluid correlations and similarity equations. The numerical computation of the model is carried out and elaborated the results via graphs. From the analysis, it concluded that the axial and transverse motion of the nanofluid drops abruptly against the stronger velocity and magnetic field effects. The thermal transport in the nanofluid improves due to the influence of thermal radiations, viscous dissipation, magnetic field and convectively heated surface. Further, local thermal transport intensifies for Rd and B_i_ parameters. Moreover, high thermal transport mechanism exists in the nanofluid which allow it for many industrial applications.

## Data Availability

The authors declared no additional data for this manuscript.

## List of symbols

$${\widehat{\rho }}_{nf}$$: Al_2_O_3_-H_2_O density
$\phi$: Volumetric fraction of Al_2_O_3_ nanoparticles
$${\widehat{\rho }}_{p}$$: Al_2_O_3_ density
$${\widehat{\rho }}_{f}$$: H_2_O density
${\widehat{\mu }}_{nf}$: Dynamic viscosity of Al_2_O_3_-H_2_O
${\widehat{\mu }}_{f}$: Dynamic viscosity of H_2_O
${\widehat{k}}_{nf}$: Thermal conductivity of Al_2_O_3_-H_2_O
${\widehat{k}}_{f}$: Thermal conductivity of H_2_O
Re_b_: Reynolds number due to particles brownian motion
$${\widehat{d}}_{p}$$: Nanoparticles diameter
${\widehat{k}}_{b}$: Stefan Boltzmann coefficient
M^*^: Molecular weight
N^*^: Avogadro number
$${\widehat{d}}_{f}$$: Molecular diameter
${\widehat{\sigma }}_{nf}$: Electrical conductivity of Al_2_O_3_-H_2_O
${\widehat{\sigma }}_{p}$: Electrical conductivity of Al_2_O_3_
${\widehat{\sigma }}_{f}$: Electrical conductivity of H_2_O
$${\widehat{C}}_{p}$$: Heat capacity
($\widehat{u},\widehat{v},\widehat{w}$): Velocity components along (x, y, z) directions
$$\widehat{g}$$: Gravitational acceleration
$$\stackrel{\smile}{T}$$: Temperature
$$({\stackrel{\smile}{u}}_{w}, {\stackrel{\smile}{v}}_{w})$$: Stretching velocities
${\stackrel{\smile}{h}}_{f}$: Thermal transfer coefficient
${\lambda }_{0}$: Velocity slip coefficient
$${\stackrel{\smile}{\lambda }}_{0},\stackrel{\smile}{c}$$: Constants
$$M$$: Hartmann number
$\lambda$: Mixed convection parameter
$Rd$: Thermal radiation number
$$Ec$$: Eckert number
$$Pr$$: Prandtl number
$$\stackrel{\smile}{S}$$: Velocity slip
$${B}_{i}$$: Biot number
$c$: Stretching ratio parameter
$R{e}_{x}$: Local Reynold number
$$N{u}_{x}$$: Nusselt number
${^{\prime}}$: First order derivative
$${^{\prime}}{^{\prime}}$$: Double derivative
${^{\prime}}{^{\prime}}{^{\prime}}$: Third order derivative
